# Non−Invasive Assessment, Classification, and Prediction of Biophysical Parameters Using Reflectance Hyperspectroscopy

**DOI:** 10.3390/plants12132526

**Published:** 2023-07-02

**Authors:** Renan Falcioni, Glaucio Leboso Alemparte Abrantes dos Santos, Luis Guilherme Teixeira Crusiol, Werner Camargos Antunes, Marcelo Luiz Chicati, Roney Berti de Oliveira, José A. M. Demattê, Marcos Rafael Nanni

**Affiliations:** 1 Department of Agronomy, State University of Maringá, Av. Colombo, 5790, Maringá 87020-900, Paraná, Brazil; glaucioalemparte@gmail.com (G.L.A.A.d.S.); wcantunes@uem.br (W.C.A.); mlchicati@uem.br (M.L.C.); rboliveira@uem.br (R.B.d.O.); mrnanni@uem.br (M.R.N.); 2 Embrapa Soja (National Soybean Research Center—Brazilian Agricultural Research Corporation), Rodovia Carlos João Strass, s/nº, Distrito de Warta, Londrina 86001-970, Paraná, Brazil; luis.crusiol@colaborador.embrapa.br; 3 Department of Soil Science, Luiz de Queiroz College of Agriculture, University of São Paulo, Av. Pádua Dias, 11, Piracicaba 13418-260, São Paulo, Brazil; jamdemat@usp.br

**Keywords:** algorithms, biophysical parameters, gibberellins, growth and development, partial least square regression, plant phenotyping, vegetation indices, wavelengths

## Abstract

Hyperspectral technology offers significant potential for non-invasive monitoring and prediction of morphological parameters in plants. In this study, UV−VIS−NIR−SWIR reflectance hyperspectral data were collected from *Nicotiana tabacum* L. plants using a spectroradiometer. These plants were grown under different light and gibberellic acid (GA_3_) concentrations. Through spectroscopy and multivariate analyses, key growth parameters, such as height, leaf area, energy yield, and biomass, were effectively evaluated based on the interaction of light with leaf structures. The shortwave infrared (SWIR) bands, specifically SWIR1 and SWIR2, showed the strongest correlations with these growth parameters. When classifying tobacco plants grown under different GA_3_ concentrations in greenhouses, artificial intelligence (AI) and machine learning (ML) algorithms were employed, achieving an average accuracy of over 99.1% using neural network (NN) and gradient boosting (GB) algorithms. Among the 34 tested vegetation indices, the photochemical reflectance index (PRI) demonstrated the strongest correlations with all evaluated plant phenotypes. Partial least squares regression (PLSR) models effectively predicted morphological attributes, with R^2^_CV_ values ranging from 0.81 to 0.87 and RPD_P_ values exceeding 2.09 for all parameters. Based on Pearson’s coefficient XYZ interpolations and HVI algorithms, the NIR−SWIR band combination proved the most effective for predicting height and leaf area, while VIS−NIR was optimal for optimal energy yield, and VIS−VIS was best for predicting biomass. To further corroborate these findings, the SWIR bands for certain morphological characteristic wavelengths selected with *s*−PLS were most significant for SWIR1 and SWIR2, while *i*−PLS showed a more uniform distribution in VIS−NIR−SWIR bands. Therefore, SWIR hyperspectral bands provide valuable insights into developing alternative bands for remote sensing measurements to estimate plant morphological parameters. These findings underscore the potential of remote sensing technology for rapid, accurate, and non-invasive monitoring within stationary high-throughput phenotyping systems in greenhouses. These insights align with advancements in digital and precision technology, indicating a promising future for research and innovation in this field.

## 1. Introduction

The global population is projected to exceed 9.8 billion by 2050, necessitating ever-increasing demand for efficient plant phenotyping and sustainable agricultural practices [1]. This escalating pressure requires innovative approaches in plant science to optimize crop growth and resource allocation [2]. High-throughput characterization, integrating remote sensing technologies [3], plant breeding [4], and computational intelligence [5], has emerged as a promising solution for enhancing phenotypic evaluation and, ultimately, crop performance in diverse environments [6]. Hyperspectral remote sensing, in particular, has gained prominence as a powerful tool for non-destructive and non-invasive evaluation of numerous plant physiological [7], biochemical [8,9], and morphological properties [10,11]. This capability facilitates the prediction of essential plant development aspects, such as size, growth cycle, and productivity, which are critical for the success of any agricultural production system [12,13,14].

Tobacco (*Nicotiana tabacum* L.), an economically significant crop grown worldwide, is an ideal model for research and studies [12,15,16]. The growth and development of tobacco plants are strongly influenced by various environmental interactions, such as light quality and intensity, as well as endogenous factors such as gibberellin [17], a key plant hormone regulating several aspects of growth and development [18], including stem elongation [17], leaf expansion [19], increased yield biomass [20], and flowering [21]. Investigating the interactions between light environments and gibberellin levels is crucial for enhancing tobacco cultivation practices and optimizing resource allocation. Applied studies for modeling these interactions for productivity in greenhouses [17,20] are also significant. With tobacco cultivation in southern Brazil experiencing rapid growth, evaluating biophysical attributes highly correlated with hyperspectral reflectance offers opportunities for more efficient, sustainable, and cost-effective analysis of this crop in greenhouses or indoor environments through precision and digital agriculture.

Reflectance hyperspectral remote sensing facilitates the acquisition of continuous and high-resolution spectral data, enabling detailed characterization of plant properties at different growth stages [22,23,24]. Recent advancements in hyperspectral technology have led to the development of more efficient algorithms for complex dataset analysis, allowing for the extraction of relevant plant traits and their correlation with specific phenotypic parameters [5,6,24]. Consequently, this technology provides a robust means for investigating the interplay between light conditions, gibberellin levels, and tobacco plant growth characteristics [12,25].

Many studies have demonstrated that applying hyperspectral remote sensing to plant phenotyping has numerous advantages [14,26]. This approach enables scientists and researchers to gain valuable insights into the growth and development of crops without causing damage, using toxic reagents, incurring high equipment costs, or performing destructive analyses on plants [27,28]. In addition, hyperspectral remote sensing has been used to predict and monitor various plant characteristics, including height, leaf area, pigment concentrations, water status, lignin and cellulose contents, biomass, and grain yield [17,20]. These parameters are essential for understanding plant growth dynamics and sustainability practices [29].

In recent years, the significance of artificial intelligence (AI) and classification algorithms in the analysis of remote sensing data has become increasingly evident [7,30,31]. Advanced machine algorithms and artificial intelligence facilitate the efficient processing of large and complex datasets, allowing researchers to extract meaningful information from the vast amounts of spectral data generated by reflectance hyperspectral data [7,32,33]. Accordingly, as shown in Mao et al. (2022) [34] and Matysiak et al. (2022) [35], machine learning and AI techniques have been employed to develop accurate and robust classification models, which can be used to predict numerous plant characteristics and growth parameters [4,36,37,38]. For instance, higher prediction and classification accuracies have been achieved in wheat, soybean, coffee, carrot, radish, and lettuce plants [3,10,32].

Vegetation indices, derived from spectral reflectance measurements, have been extensively employed in remote sensing applications for monitoring plant health, growth, and productivity [16,39,40,41]. These indices are particularly useful in spectroscopy analyses, as they can help identify specific wavelengths or range bands most strongly associated with particular plant morphological parameters [11,42,43]. For example, the selection of appropriate wavelengths and the development of customized vegetation indices can significantly improve the accuracy and efficiency of hyperspectral remote sensing applications in agriculture and environmental studies [7,20,37].

Our study aims to integrate advanced computational intelligence, classification algorithms, and customized vegetation indices to extract valuable information from UV−VIS−NIR−SWIR hyperspectral data on plant growth in greenhouses. We hypothesize that this approach will enable non-destructive assessment, classification, and prediction of morphological parameters, such as height, leaf area, energy yield, and biomass of tobacco plants, with high accuracy and precision. The objective is to demonstrate that reflectance hyperspectral remote sensing technology can provide alternative, rapid methods for estimating plant attributes.

## 2. Results

### 2.1. Variance and Descriptive Analyses-Based Efficiency Parameters of Tobacco Plants

**Figure 1:**
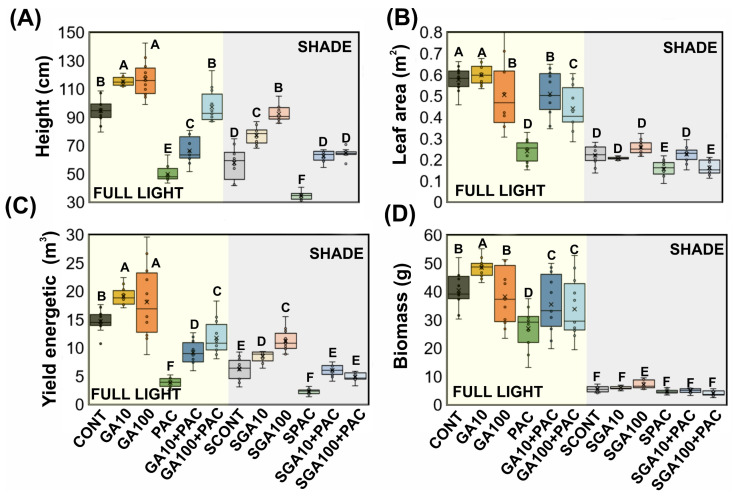
Box plot of the morphological and efficiency parameters in *Nicotiana tabacum* L. leaves of plants grown in high irradiance (yellow; full light) and low light (grey; 8.5% of full light; shade) environments and submitted to different gibberellin (gibberellic acid–GA_3_) concentration. (**A**) Height (cm). (**B**) Leaf area (m^2^). (**C**) Yield energetic (m^3^). (**D**) Biomass (g). CONT (Control; full light); GA10 (10 µM GA_3_; full light); GA100 (100 µM GA_3_; full light); PAC (50 mg L^−1^ of paclobutrazol; full light); GA10P (combined GA_3_ 10 µM + PAC; full light); GA100P (combined GA_3_ 100 µM + PAC; full light); SCONT (Control; shade); SGA10 (10 µM GA_3_; shade); SGA100 (100 µM GA_3_; shade); SPAC (50 mg L^−1^ of paclobutrazol; shade); SGA10P (combined GA_3_ 10 µM + PAC; shade); SGA100P (combined GA_3_ 100 µM + PAC; shade). Different letters above the box denote significant differences according to Duncan’s test (*p* < 0.001). Mean ± SE. (*n* = 144).

The morphological attributes relevant to the phenotyping of *Nicotiana tabacum* L. leaves were evaluated in plants. These plants were cultivated under high irradiance (full light) and low light (8.5% of full light; shade) conditions and under different gibberellin treatments. The results presented in Table 1 and Figure 1 display significant differences (*p* <0.001) in height (cm), leaf area (m^2^), yield energetic (LAD; m^3^), and biomass (g) values. In addition, Table 1 and Figure 1 also include the coefficient of variation (CV%) of the parameters for twelve gibberellin levels under distinct light conditions. The CV values ranged from 33.5 to 82.6%, with all four parameters classified as very high. The results suggest a significant degree of variation in the studied plant characteristics.

Higher concentrations of GA_3_ induced an increase in light capture efficiency parameters and plant growth, both under high and low light availability conditions (Figure 1). On the other hand, applications of PAC under the plants reduced growth by more than (≈86%) in both full or low light conditions in greenhouses. However, when GAs were added to the PAC treatments, the plants partially reversed their growth and yield conditions toward dry matter accumulation. In general, higher concentrations of GA_3_ (100 µM) and GA100+PAC (Figure 1A,D) and increments valuable of parameters (Figure 1B,C) were observed (Figure 1; light vs. GAs).

### 2.2. Leaf Hyperspectral Reflectance

**Figure 2:**
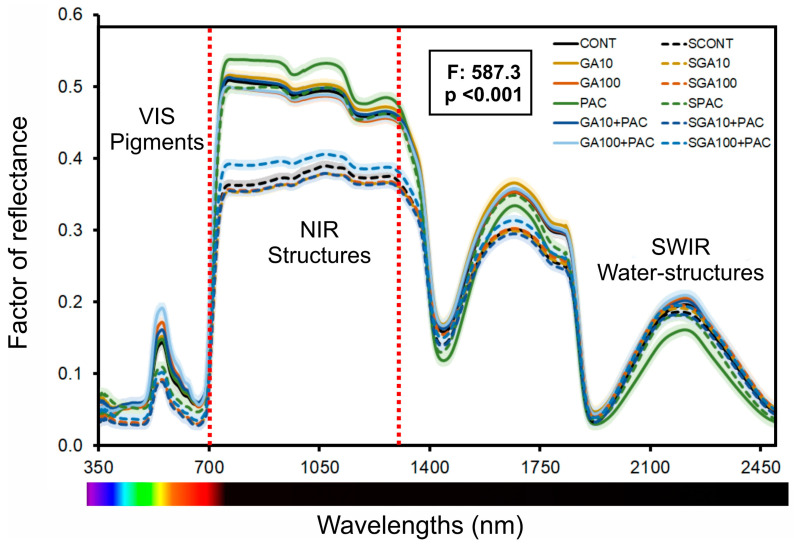
The factor of reflectance was calculated for spectral leaf curves spanning from 350 to 2500 nm in *Nicotiana tabacum* L. leaves. The plants were cultivated under two different light conditions, high irradiance (full light) and low light (8.5% of full light), and submitted to different concentrations of gibberellic acid (GA_3_). The determination of the accurate inflection points at 700 and 1300 nm was conducted (red dotted lines). Each repetition was generated by calculating the mean of measurements taken for leaves. For abbreviations pertaining to other treatments, see Figure 1. F-test (*p* < 0.001). (*n* = 144). To enhance clarity, the standard error was omitted.

The leaf photosynthetically active radiation (PAR) reflectance in vivo (350–700 nm) exhibited the highest values in individuals treated with GA100P, reaching a peak of approximately 23% at the green wavelength (550 nm). In contrast, control plants grown under high irradiance conditions displayed the lowest peak reflectance values (*p* < 0.001) (Figure 2). Comparing the GA_3_ regimes to their corresponding control plants grown in full light, there were minor differences (<5%) in reflectance values within the blue and red bands. However, notable changes were observed when comparing shaded environments, with an approximate increase of ≈37% between these distinct conditions. Generally, plants grown under low light conditions demonstrated a decrease in their peak reflectance value at 550 nm (Figure 2). In comparison to plants grown under full light, higher reflectance values were observed in control (14.7%) and GA10P-treated (12.2%) plants, while lower values were noted in PAC-treated plants (7.8%) at 550 nm. Among all treatments, only control plants grown under low light conditions exhibited higher reflectance values in the blue or red regions of the spectrum. PAC-treated plants displayed comparable reflectance values to their GA_3_-treated corresponding in the blue or red spectral bands, both in high irradiance and low light-grown plants.

In the NIR spectrum ranging from 700 to 1300 nm, distinct patterns were observed in the hyperspectral curves of leaf development under light conditions (Figure 2). Leaves grown in high light availability environments showed an average increase of 42.5% in reflectance within this spectral band compared to shade-grown plants, except for SPAC, which demonstrated similar increases to full light-grown plants. In the SWIR region (1500–2500 nm), characteristic points of water absorption were observed at 1450, 1680, 1840, 1930, and 2490 nm, and three other peaks related to structural components (1192, 1678, and 2220 nm), including cell walls, cellulose, lignin, pectin, and other compounds (1270, 1797, and 2130 nm) associated with phenolic compounds, were evident in the hyperspectral curves (Figure 2). Furthermore, parameters such as photochemical−photosynthetic efficiency and phenotyping were correlated with the bands close to 1500, 1600, and 1810 nm in the SWIR band for photochemical efficiency. These spectral patterns were associated with leaf development, with higher reflectance factors observed for leaves with more developed cells in the mesophyll and lower values for less developed cells (see Figure 2; SWIR water structures).

### 2.3. Clustering and Correlation Analysis Using PCA

**Figure 3:**
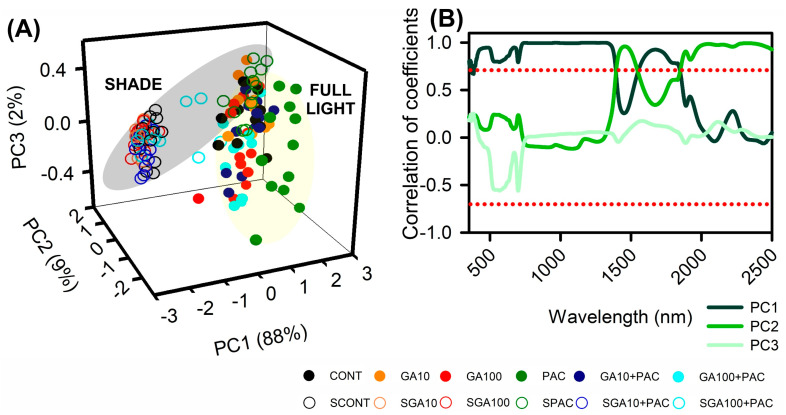
Principal component analyses (PCAs) of spectral leaf reflectance curves ranging from 350 to 2500 nm in *Nicotiana tabacum* leaves from plants cultivated in high irradiance (yellow; full light) and low light (grey; 8.5% of full light) environments and submitted to different GA_3_ concentrations. (**A**) 3D plot of the PCA scores for the PC1, PC2, and PC3 of hyperspectroscopy data. (**B**) Correlation of coefficients with three principal components (dark to light green lines; PC1, PC2, and PC3). The red line represents −0.70 and +0.70 correlation coefficients. For detailed information on treatments and their abbreviation, please refer to Figure 1. (*n* = 144).

Figure 3 displays the relationship between wavelengths and principal components (PCs) in tobacco plants that were grown under different levels of gibberellin treatment and varying light conditions. The hyperspectral curves exhibit significant correlations (*p* < 0.001) between PCs (PC1–PC3) and distinctive environmental factors, highlighting the potential of hyperspectral analysis for clustering tobacco plants based on their morphological and yield parameters (Figure 3A). The PCA explained 99% of the total variance, with PC1, PC2, and PC3 accounting for 88%, 9%, and 2%, respectively, for the spectral range of 350 to 2500 nm.

The spectral curves demonstrate strong correlations throughout the full spectrum, indicating that spectral analysis can provide valuable insights into the alterations in leaf properties under full light and gibberellin treatments. Specifically, changes in the NIR and SWIR regions of the hyperspectral curves (350–2500 nm) strongly correlate with the effects of environmental factors on plant growth and phenotyping for height, leaf area, yield energetic, and biomass (Figure 3), resulting in two distinct clusters.

Figure 3B shows that the VIS spectrum region near the blue (435 nm), green (555 nm), and red (650 nm) bands has increased correlation coefficient values for the first components, while minor correlations are observed for the second and third components, indicating good differentiation properties among tobacco plants. Additionally, near-infrared and shortwave-infrared bands (760–850 nm, 880–1000 nm; 1450–1600, and 1800–2100) exhibit strong correlations for the separation of treatments, highlighting the potential of hyperspectral analysis for assessing the effects of environmental factors on tobacco plant growth and phenotyping for the second and third components.

### 2.4. Selected Wavelength Cluster Heatmap

**Figure 4:**
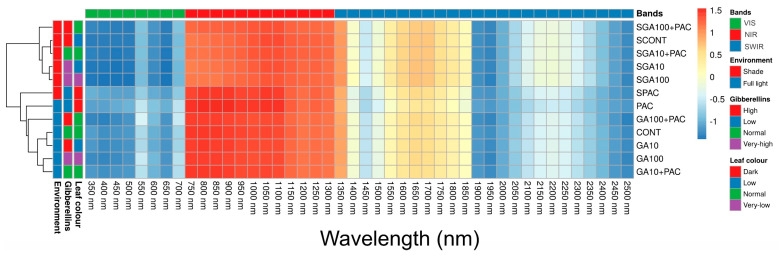
The cluster heatmap displayed the correlation between the hyperspectral bands for *Nicotiana tabacum* L. leaves from plants cultivated in high irradiance (full light) and low light (8.5% full light) environments and submitted to different GA_3_ concentrations. The correlations are arranged by wavelength bands (VIS, NIR, SWIR), environment (sun, shade), gibberellin regimes (low, normal, high, very high), and leaf colors (very low, low, normal, dark). Positive correlations are shown in red, and negative correlations are shown in blue (Z-score, *p* < 0.001). For detailed information on treatments and their abbreviation, please refer to Figure 1. (*n* = 144).

The relationship between different environmental conditions, gibberellin levels, leaf colors, and hyperspectral data wavelengths in tobacco plants was examined using cluster heatmap analysis, as shown in Figure 4. The colors on the cluster heatmap indicate the Z-score between the hyperspectral values and the morphological and growth conditions of the plants, which are strongly associated with components such as pigments and structure. In this sense, Z-scores were employed to represent the variability of the data, with red colors displaying a correlation slope of approximately 1.5, blue colors indicating a slope of approximately −1.5, and light colors representing a weak association with values of approximately 0.

A darker shade of blue for a specific wavelength band indicated a higher GA_3_ concentration of a particular environmental condition, which could be distinguished clearly. Gibberellic acid regimes, which directly impact plant efficiency and morphological changes, were strongly associated with bands in the near-infrared (NIR) region and, to a lesser extent, in the visible (VIS) region. On the other hand, the bands between 1400 and 1450 nm were negatively correlated with components in all plants (PCA vs. light), while those between 1900–1950 nm and 2450–2500 nm showed a strong interaction with the increment of gibberellin levels in the plants (Figure 4). Furthermore, the data demonstrated that in all NIR−SWIR bands, there were strong and significant changes in the spectra associated with plants grown in full light and shade environments, with plants grown in full light demonstrating a greater impact of structural changes compared to those grown in shaded environments, independent of the concentration of GA_3_ (Figure 4, see 750–1300 nm).

### 2.5. Classification of Morphological and Phenotyping for Plant Growth with Machine Learning and Intelligence Algorithms Models

**Figure 5:**
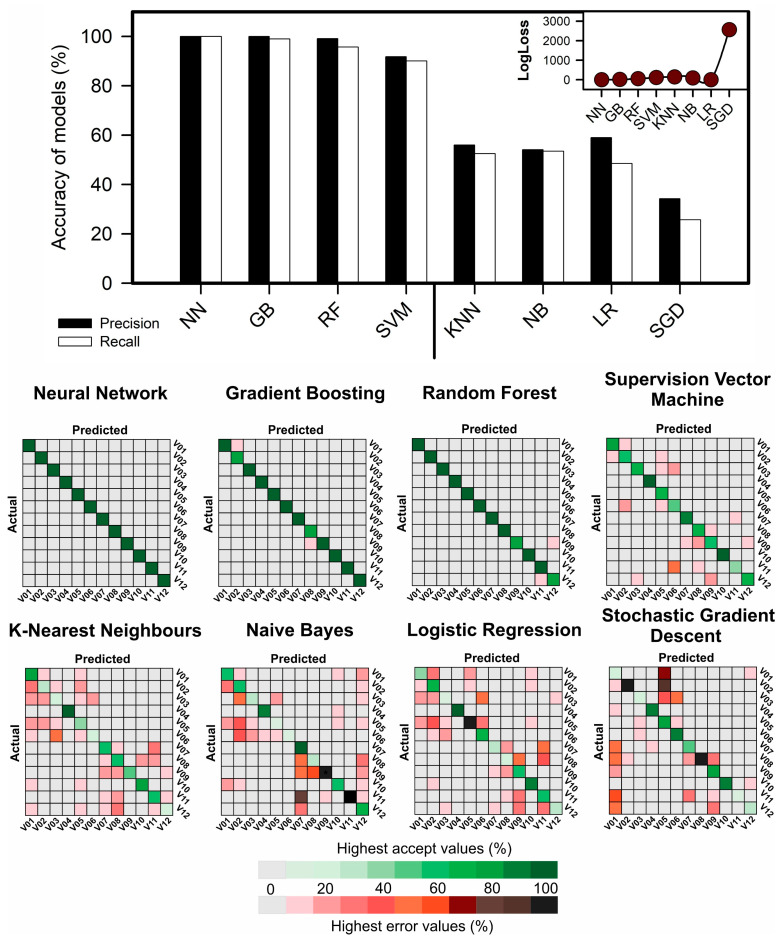
Evaluation of models for precision and recall for 8 machine learning and artificial intelligence algorithms (AIAs) for *Nicotiana tabacum* L. leaves from plants cultivated in high irradiance (full light) and low light (8.5% of full light) environments and submitted to different GA_3_ concentrations. Confusion matrix for neural network (NN), gradient boosting (GB), random forest (RF), supervision vector machine (SVM), K-nearest neighbors (KNN), naive Bayes (NB), logistic regression (LR), stochastic gradient descent (SGD). Inset shows LogLoss for error accumulation in models for classification. Boxes show overall accuracy/precision for correct classification (accepted in green) and mistake (error in red). A total of 100 training samples and 44 validation samples were used. For abbreviations V01−12, which indicate the order of treatments, see Figure 1. (*n* = 144).

The assessment of morphological and phenotyping characteristics using VIS−NIR−SWIR hyperspectral data is presented in Figure 5. Eight machine learning algorithms were utilized, including neural network (NN), gradient boosting (GB), random forest (RF), support vector machine (SVM), K-nearest neighbors (KNN), naive Bayes (NB), logistic regression (LR), and stochastic gradient descent (SGD), each showing varying levels of performance ranging from low to high accuracy. To assess the performance of these algorithms, three accuracy parameters were employed, namely, correct classification (CC), F-score, and kappa coefficient. The variance analysis depicted in Figure 5 demonstrates a significant interaction between the morphological inputs and the machine learning algorithms tested for F-score, with a significant difference for ML techniques concerning CC and Kappa.

Regarding the classification of tobacco plants under different GA_3_ regimes and light environments, the neural network (NN) and gradient boosting (GB) algorithms demonstrated the highest performance, with an average accuracy of over 99% (Figure 5). The study used a total of 100 training samples and 44 validation samples. The random forest (RF) and support vector machine (SVM) algorithms also had relatively high accuracy, averaging approximately 88% to 95%, with similar values for the training and testing datasets. In contrast, the K-nearest neighbors (KNN), naive Bayes (NB), logistic regression (LR), and stochastic gradient descent (SGD) algorithms had higher error rates based on the F1-test, Kappa, and accuracy performance (Figure 5). These findings suggest that both the complete range of bands and individual wavelength values have the potential for accurate classification when applied with artificial intelligence techniques (Figure 5).

### 2.6. Vegetation Indices for Morphological and Phenotyping Monitoring Parameters

**Figure 6:**
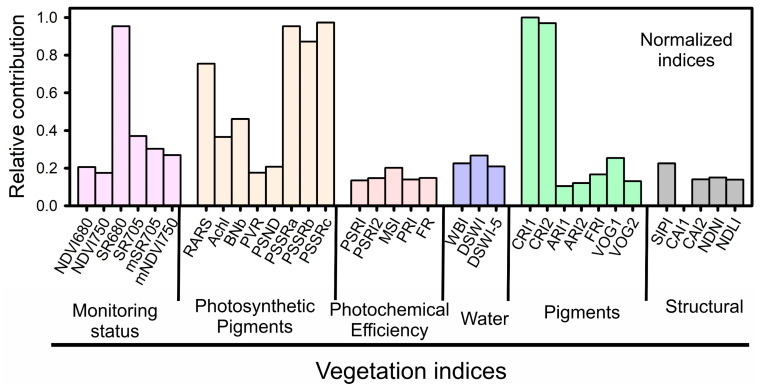
Relative contribution of narrowbands (VIs) to total variability for spectral leaf reflectance data from 350 to 2500 nm in *Nicotiana tabacum* L. leaves from plants cultivated in high irradiance (full light) and low light (8.5% of full light) environments and submitted to different GA_3_ concentrations. For abbreviations of the vegetation indices, see Table S1. Each vegetation index was separated for more correlation of specific differences in biochemical and structural compounds. Monitoring status (pink bars), photosynthetic pigments (yellow bars), photochemical efficiency (orange bars), water (blue bars), pigments (green bars), and structural (grey bars).

Several vegetation indices (VIs) were evaluated for their effectiveness in monitoring plant status, photosynthetic pigment assessment, and structural parameters. The simple ratio index ρ680 (SR680) emerged as the most effective VI for monitoring plant status. For photosynthetic pigment assessment, the strongest correlations were observed with the ratio analysis of reflectance spectra (RARS), pigment-specific simple ratio Chl a (PSSRa), pigment-specific simple ratio Chl b (PSSRb), and pigment-specific simple ratio (PSSRc) (Figure 6 and Figure S1). These indices were found to be effective in assessing the levels of chlorophyll *a*, chlorophyll *b*, and carotenoids in the plants. Additionally, carotenoid reflectance index 1 (CRI1) and carotenoid reflectance index 2 (CRI2) emerged as the most suitable indices for pigment monitoring (Figure 6 and Figure S1). These indices were found to be effective in assessing the levels of carotenoids in the plants (Table S1). Finally, the structurally insensitive pigment index (SIPI) was identified as the optimal index for evaluating structural parameters. This index was found to be effective in assessing the structural characteristics of the plants, such as leaf area and biomass (Figure 6; see Structural in grey).

### 2.7. Matrix of Correlations for Vegetation Indices for Monitoring Morphological Parameters

**Figure 7:**
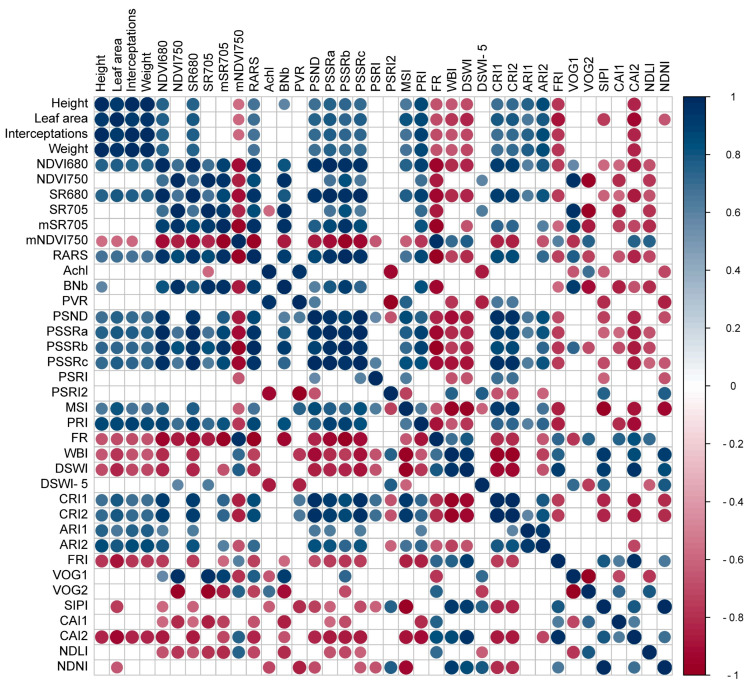
Pearson’s correlation matrix between each of the morphological, efficiency, and vegetation indices parameters valuable for *Nicotiana tabacum* L. leaves from plants cultivated in high irradiance (full light) and low light (8.5% of full light) environments and submitted to different GA_3_ concentrations. (*p* < 0.001) Abbreviations are described in Table S1.

The correlation matrix of vegetation indices for monitoring morphological parameters revealed strong relationships between the indices and their corresponding parameters, as well as some negative correlations. The photochemical reflectance index (PRI) demonstrated the highest and strongest correlations (r = 0.87, 0.87, 0.88, 0.86; *p* < 0.001) for monitoring plant morphological parameters, including height, leaf area, yield energetic, and biomass, respectively (Figure 7). However, the simple ratio index ρ680 (SR680) showed a strong positive correlation (r = 0.75) with plant height. The energy yield parameter was strongly correlated with the NDVI680 (r = 0.77) and SR680 (r = 0.78) indices. However, some indices showed negative correlations with certain parameters. For instance, the PSSRa index had a negative correlation with chlorophyll *b* content (r = −0.64), while the PSSRb and PSSRc indices exhibited negative correlations with chlorophyll *a* content (r = −0.58 and r = −0.65, respectively). In addition, the normalized difference vegetation index750 (NDVI750) showed a low correlation with plant height (r = 0.41). Regarding the assessment of photosynthetic pigments, the ratio analysis of reflectance spectra (RARS), pigment-specific simple ratio Chl a (PSSRa), pigment-specific simple ratio Chl b (PSSRb), and pigment-specific simple ratio (PSSRc) indices yielded the strongest correlations. Among the pigments, the carotenoid reflectance index 1 (CRI1) and carotenoid reflectance index 2 (CRI2) showed the strongest correlations, while the structurally insensitive pigment index (SIPI) was found to be the best index for evaluating structural parameters. In this way, the cellulose absorption index (CAI) demonstrated the highest and negative correlations (r = −0.83, −0.92, −0.84, −0.83; *p* < 0.001) for monitoring the structure and ultrastructure of cell components and leaf tissues for phenotyping such height, leaf area, yield energetic, and biomass, respectively (Figure 7). Overall, these findings suggest that a combination of different vegetation indices can be utilized to accurately monitor morphological and photosynthetic parameters in plants. The identified correlations provide a basis for developing a comprehensive monitoring strategy that incorporates a suite of vegetation indices tailored to the specific monitoring needs of the plant species of interest (Figure 7).

### 2.8. Prediction of Morphological and Efficiency Phenotyping Parameters

The statistical metrics of the PLSR models for height, leaf area, yield energetic, and biomass parameters obtained from the calibration (Cal) and cross-validation (Cva) methods are presented in Table 2. The analysis of hyperspectral data collected from 350 to 2500 nm indicated a clear difference in behavior between the Cal and Cva phases. The four best results had excellent calibration scores (>0.83 and >2.45, respectively) for the R^2^_C_ and RPD_C_ metrics. The full hyperspectral data (350–2500 nm) provided the best R^2^_CV_ and RPD_CV_ metrics (R^2^_CV_ > 0.81, RPD_CV_ > 2.3) (Table 2).

Models containing 1 to 3 factors were considered the most appropriate for capturing the relationship between predictor variables (reflectance) and predicted variables (tobacco growth and phenotyping parameters), as shown in Table 2. The R^2^_CV_ values indicated very good models (0.81–0.87) for height, leaf area, yield energetic, and biomass, while the RPD_P_ values indicated excellent models (>2.09) for all variables evaluated. The RMSE_CV_ values were similar or slightly smaller than RMSE_P_, and the bias was close to zero for all parameters.

To evaluate the predictive capacity of the PLSR models for the four proposed parameters, an independent dataset of hyperspectral data was used. For example, a model with 1 factor for yield energetic and 3 factors for height, leaf area, and biomass was considered excellent for predicting tobacco parameters.

**Figure 8:**
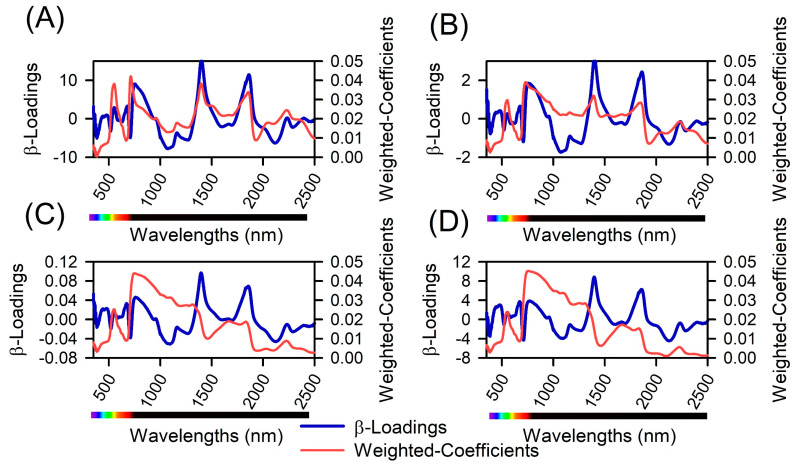
β−loadings and weighted-coefficient method-based reflectance hyperspectral data between 350 and 2500 nm for *Nicotiana tabacum* L. leaves from plants cultivated in high irradiance (full light) and low light (8.5% of full light) environments and submitted to different GA_3_ concentrations. (**A**) Height (cm). (**B**) Leaf area (m^2^). (**C**) Yield energetic (m^3^). (**D**) Biomass (g).

The β−loadings and weighted coefficients of the PLSR model are illustrated in Figure 8, which identifies the peaks and valleys with significant impacts on the prediction model construction. These features were distributed evenly across all spectra (VIS−NIR−SWIR). To create the PLSR models, RC and VIP values were employed, and the models were based on 5 to 12 wavelengths (peaks and valleys). The wavelengths with the highest coefficients were found to be near 400 nm (violet), 435 nm (blue), 555 nm (green), 672 nm (red), 700–750 nm (red edge), 1350 nm (NIR), 1460 nm (SWIR), 1950 nm (SWIR), and 2200 nm (SWIR). These wavelengths were found to effectively contribute to high precision in predicting parameters such as plant height (Figure 8A), leaf area (Figure 8B), yield energetic (Figure 8C), and biomass (Figure 8D).

### 2.9. XYZ Interpolates Pearson’s Coefficient by Morphological and Efficiency Parameters

**Figure 9:**
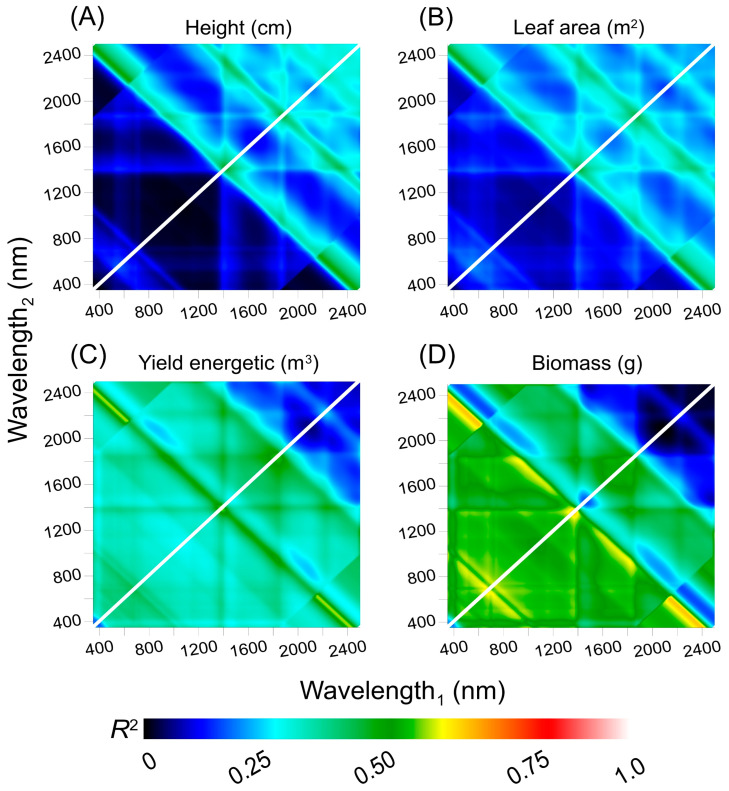
Count plot map by XYZ interpolates Pearson’s coefficient of correlation by inverse distance to a power math between morphological and efficiency parameters and wavelengths_1_ vs. wavelength_2_ for 350 to 2500 nm. (**A**) Height (cm). (**B**) Leaf area (m^2^). (**C**) Yield energetic (m^3^). (**D**) Biomass (g). White line, correlation 1:1. The displayed color gradient from dark blue to light red indicates an increase in associations.

Figure 9 presents a count plot map that displays the correlation coefficients (R^2^) obtained from the linear regression between each parameter and Pearson correlation. Those coefficients were interpolated using combinations of two hyperspectral bands and reflectance hyperspectral sensors. The full spectra (350–2500 nm) from the hyperspectral sensors were analyzed at this stage, which improved the prediction of parameters. The analysis revealed that the NIR/SWIR band combination was the most effective for predicting height (Figure 9A) and leaf area (Figure 9B), whereas the VIS−NIR combination was the best combination for predicting yield energetic (Figure 9C). Meanwhile, the VIS−VIS combination was optimal for predicting biomass (Figure 9D) when using interpolation between wavelength_1_ and wavelength_2_ (Figure 9). When using hyperspectral sensors, the VIS/VIS band combination exhibited the highest values of R^2^ for the same spectral interval.

Moreover, the results demonstrated that the R^2^ value addressing the correlation between the light environment and gibberellin levels for each hyperspectral curve depended on the most efficient method for morphological and phenotyping attributes, depending on the specific bands used to monitor the key parameter. Additionally, the shortwave infrared spectrum featured numerous band combinations with high correlations (>0.61) (Figure 9).

### 2.10. Selection of Variables by PLS Algorithms and Hyperspectral Vegetation Index

**Figure 10:**
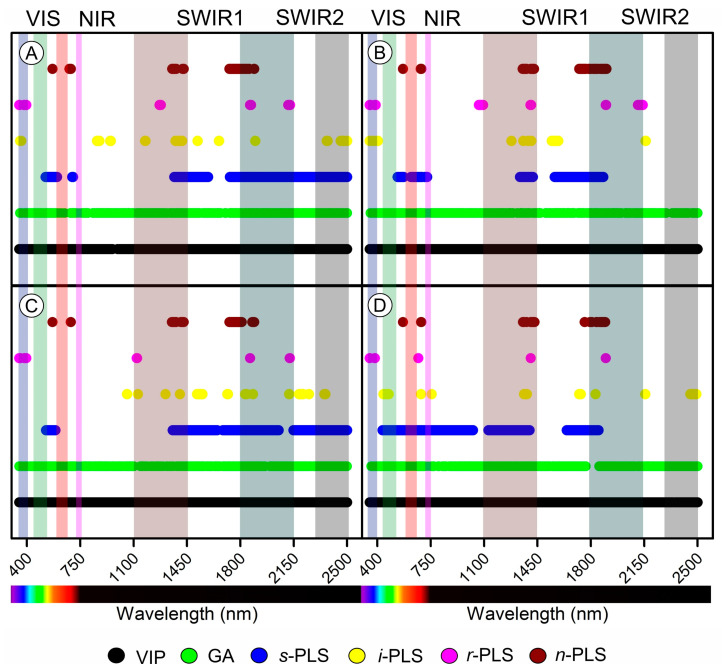
The most responsive variables were selected wavelength range of 350−2500 nm by the VIP, GA, *s*−PLS, *i*−PLS, *r*−PLS, and *n*−PLS algorithms for tobacco plant growth variables. (**A**) Height (cm). (**B**) Leaf area (m^2^). (**C**) Yield energetic (m^3^). (**D**) Biomass (g).

The effectiveness of six algorithms was assessed in terms of their capacity to identify the most reactive wavelengths within the 350 to 2500 nm range. These algorithms included VIP, GA, *s*−PLS, *i*−PLS, *r*−PLS, and *n*−PLS (Figure 10). All algorithms selected wavelengths in the VIS, NIR, SWIR1, and SWIR2 bands. However, the VIP and GA algorithms were unable to discriminate between a few wavelengths and essentially selected almost all of the 2151 bands collected during the performance for the generated phenotypic classification models based on height (Figure 10A), leaf area (Figure 10B), yield energetic (Figure 10C), and biomass (Figure 10D).

In contrast, the models generated by *s*−PLSR (dots in blue) were most significant for SWIR1 and SWIR2, with few shared wavelengths in the VIS (570 and 630 nm). The wavelengths of *i*−PLS exhibited a more uniform distribution, although they presented between 22 and 33 selected wavelengths (Figure 10A−D, yellow dots).

Conversely, *r*−PLS and *n*−PLS (pink and red dots) showed the lowest levels of wavelength selection but encompassed the entire spectrum (VIS−NIR−SWIR). This analysis highlights the potential of the *s*−PLS, *i*−PLS, *r*−PLS, and *n*−PLS algorithms in identifying the most responsive wavelengths for assessing phenotypic parameters of tobacco plants and paves the way for more efficient and accurate monitoring of plant growth (Figure 10). Therefore, height, leaf area, yield energetic, and biomass should be better characterized by SWIR bands.

**Figure 11:**
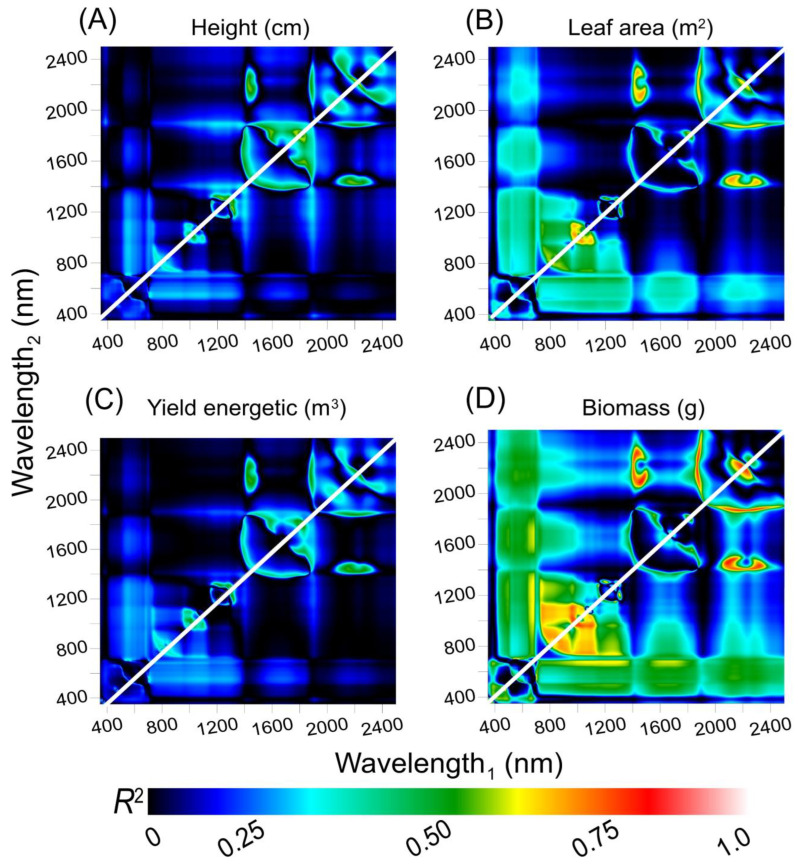
The count plot map displays the coefficient of correlation (R^2^) obtained from linear regression analysis between morphological and phenotyping parameters and wavelengths_1_ vs. wavelength_2_ in the range of 350 to 2500 nm. (**A**) Height (cm). (**B**) Leaf area (m^2^). (**C**) Yield energetic (m^3^). (**D**) Biomass (g). White line, correlation 1:1. The displayed color gradient from dark blue to light red indicates an increase in associations.

On the other hand, the calculated hyperspectral vegetation indices (HVI; Figure 11) exhibited a low correlation for VIS−VIS and VIS−NIR combinations, as well as an intermediate VIS−SWIR band for height (Figure 11A) and leaf area (Figure 11B). The highest correlations were found for NIR−SWIR at 1464 and 2095 nm (R^2^ = 0.78) for biomass (Figure 11D) and the SWIR−SWIR band at 2212 nm and 2229 nm (R^2^ = 0.63) for yield energetic (Figure 11C) combinations (Figure 11).

## 3. Discussion

### 3.1. Efficiency Parameters and Leaf Hyperspectral Reflectance of Tobacco Plants

The significant differences observed in height (cm), leaf area (m^2^), yield energetic (m^3^), and biomass (g) values (*p* < 0.001) under different light conditions and gibberellin treatments in the present study underscore the complex interaction of environmental and hormonal factors on the growth and productivity of *Nicotiana tabacum* L. plants (Table 1 and Figure 1) [17,44,45]. In line with prior research, our results emphasize the importance of light conditions in plant growth and development [16], as well as in the accurate estimation, classification, and prediction of phenotypic growth parameters. For example, studies by Alabadí et al. (2008) [46], Lau and Deng (2010) [47], and Kurepin and Pharis (2014) [48] reported that varying light conditions could lead to morphological and physiological acclimation in plants. This correlates with the observed changes in tobacco plants under differing light intensities in greenhouses. Thus, the prediction of biophysical and morphological attributes using hyperspectral reflectance and non-invasive methods could provide alternative monitoring techniques when large differences between plants are observed.

Our findings corroborate the well-established role of gibberellins (GAs) in promoting plant growth [48,49]. Upon application, reflectance hyperspectral data reveal increased variations in several analyzed parameters. The influence of GAs on cell elongation and division, as discussed by Biemelt et al. (2004) [50], aligns with the observed increases in height, leaf area, and biomass values in tobacco plants supplemented with GAs. For instance, GA_3_ significantly affects the reflectance factor and, consequently, growth parameters (Figure 1, Figure 2 and Figure 3). Conversely, paclobutrazol (PAC), which inhibits GA biosynthesis and suppresses plant growth [17,18,51], aligns with our observation of reduced growth in tobacco plants treated with PAC. These findings underscore the role of GAs in plant growth regulation. These characteristics subsequently alter leaf optical properties and other morphological, anatomical, biochemical, and genetic features that can be effectively quantified using reflectance hyperspectroscopy. Thus, VIS−NIR−SWIR is an effective method for understanding the interplay between gibberellin and light intensities in greenhouses for protected cultivation.

The high degree of variability in plant characteristics, as evidenced by the high coefficient of variation (CV%) values, concurs with previous research on other crop species [11,52]. This variability may be ascribed to genetic and environmental factors, as well as their interactions, as reported in [53,54]. Comprehending the sources of variability and devising strategies to optimize plant growth and productivity under different environmental conditions remains a crucial future research area for monitoring base leaf−clips in leaves.

Leaf-based hyperspectral reflectance has been recognized as a promising non-destructive technique for monitoring plant growth and development, a view supported by an expanding body of literature [43,55,56]. For example, studies by Calviño−Cancela and Martín−Herrero (2016) and Sexton et al. (2021) [57] have demonstrated the potential of hyperspectral remote sensing in assessing the physiological status of crops, consistent with our findings regarding differences in visible (VIS) bands (Figure 2 and Figure 3A,B). The distinct patterns observed in the near-infrared (NIR) and shortwave infrared (SWIR) regions of the spectrum (Figure 2) align with previous research, which has reported the potential of hyperspectral reflectance as a tool for assessing plant physiological processes and phenotyping characterization [18,51]. However, further investigation of the relationships of spectral patterns with physiological processes is crucial to enhance the accuracy and applicability of hyperspectral remote sensing in crop science and agronomy fields [11,58,59].

### 3.2. Advanced Data Analysis and Wavelength Selection for Enhanced Plant Growth Estimation

Hyperspectral data analysis, principal component analysis (PCA), and correlation coefficients were used to assess the impact of gibberellin treatments and light conditions on the phenotyping of tobacco plants (Figure 3A). These methods have been previously shown to be effective in capturing variability in plant growth and yield parameters under different quality and intensity light environments [13,60]. Our findings successfully achieved the initial objectives and corroborated the first hypotheses when they were analyzed by classification and prediction-linked biophysical parameters.

Furthermore, as shown in Figure 4 and demonstrated by Braga et al. (2021) [61], strong correlations exist between the visible (VIS), near-infrared (NIR), and shortwave infrared (SWIR) bands of the hyperspectral curves (350–2500 nm), and the morphological changes in leaves and alterations in plant growth under different light and gibberellin treatments can be successfully predicted [46,47,49]. This is in line with the findings of Ge et al. (2019) [40], who also observed strong correlations between the VIS and NIR regions for estimating plant biophysical parameters. However, our study further highlights the importance of the SWIR region in assessing the impact of environmental factors on plant growth and phenotyping, such as water status, which was evaluated using the WBI, DSWI, and DSWI−5 indices (Figure 7 and Figure S1).

In contrast to our findings, some researchers have reported that the red edge (680–750 nm) and NIR plateau (750–1300 nm) regions are more relevant for estimating plant growth parameters [15,42,62]. Although these regions have demonstrated potential in considering leaf ultrastructure and morphological variations under different GA_3_ regimes, our results indicate that the VIS spectrum near the blue (435 nm), green (535 nm), and red (662 nm) bands, along with specific intervals of the NIR region (750–860 nm, 870–1050 nm), as well as numerous SWIR 1 and 2 bands, provide better differentiation among tobacco varieties and treatments. For instance, SWIR exhibits excellent classification and predictive abilities for changes in the structure and ultrastructure of leaves in tobacco plants when gibberellin levels are increased (GA_3_) or decreased (PAC) (Figure 1, Figure 2, Figure 3 and Figure 4).

The selected wavelength cluster heatmap (Figure 4) confirms the importance of specific spectral bands for the discrimination and estimation of tobacco plant growth parameters under different environmental conditions. This finding is in agreement with the work of [63,64], who emphasized the role of certain wavelengths in detecting differences in plant phenotyping characteristics. Nevertheless, our study expands on this knowledge by identifying additional NIR intervals that can be useful for separating treatments.

The selection of variables by algorithms showcases the potential of machine learning techniques (Figure 5) to enhance the efficiency of hyperspectral data analysis in estimating plant growth and phenotyping parameters [33,65,66]. This supports the findings of Silva et al. (2017) [33], who also recognized the value of machine learning algorithms in agricultural applications. However, our study provides a more comprehensive approach, integrating PCA and AIAs and machine learning to extract meaningful information from hyperspectral data.

In conclusion, our analysis demonstrates that the combination of hyperspectral analysis, PCA, and learning algorithms is a powerful tool for studying the effects of gibberellin treatments and light conditions on the growth and phenotyping of tobacco plants (Figure 1, Figure 2, Figure 3, Figure 4 and Figure 5). The selected wavelengths provide a solid foundation for developing discriminant models that can be employed in future research and practical applications, ultimately contributing to enhanced agricultural productivity and management.

### 3.3. Morphological and Phenotypic Classification and Prediction Using Machine Learning and Artificial Intelligence Models

Morphological classification and phenotypic prediction of tobacco plants were conducted using eight machine learning algorithms: neural networks, gradient boosting, random forest, naive Bayes, K-nearest neighbors, support vector machines, logistic regression, and stochastic gradient descent (Figure 5 and Figure 6). Our findings are in agreement with previous research, indicating that neural networks and other machine learning algorithms can effectively classify and predict plant morphological traits and yield parameters based on hyperspectral data [15,31,42,67,68].

The excellent performance of neural networks and gradient boosting algorithms in our study aligns with the findings of Yoosefzadeh−Najafabadi et al. (2021) [69], who reported that deep learning models achieved superior accuracy in classifying plant species based on hyperspectral data. Furthermore, our results contribute to the growing body of literature highlighting the potential of AI and ML algorithms in enhancing the efficiency and accuracy of plant phenotyping with high precision [68,69,70].

Vegetation indices (VIs) have been widely used for monitoring plant growth, assessing pigment concentrations, and phenotyping in crop sciences [9,16,71,72]. They offer a rapid and accurate evaluation of various phenotypic traits across a range of crops. However, the specific reasons for their effectiveness in predicting certain traits or robust correlations without elucidating the underlying physiological phenomena remain unclear [33,73]. In our study, we identified several VIs effective for tracking plant status, evaluating photosynthetic pigments, and assessing structural parameters (Figure 6 and Figure 7; Table S1 and Figure S1). Although NDVI750, PVR, and PSND demonstrated reduced effectiveness, the performance of PRI is consistent with prior research on the utility of VIs in plant growth and health evaluation and pigment concentration estimation [26,58,71,74,75,76]. This includes the detection of structural alterations in carotenoids (lighter leaves with low chlorophyll concentrations) and shifts in cell wall structure and nitrogen content associated with leaf and biomass formation (cellulose, lignin, SIPI, CAI1, CAI2, NDNI, NDLI) [9,16,39,40,74,75,77,78].

The strong correlations identified between various VIs and morphological parameters in our study support the view that these indices can serve as reliable tools for monitoring plant growth and physiological status. This concurs with research by Fernandes et al. (2020) [58] and Brocks and Bareth (2018) [79], who reported strong correlations between VIs and plant attributes such as biomass, leaf area index, and pigment concentrations [38,58,79,80].

In predicting tobacco plant phenotypic parameters (Table 2), PLSR models demonstrated high precision in estimating various parameters related to plant growth and development. For instance, Crusiol et al. (2022a) [11] developed a method for monitoring water status, Coast et al. (2019) [81] focused on monitoring respiration, and Falcioni et al. (2023b) [12] examined nutritional deficiencies, among other research. Accurate yield prediction for plant height, leaf area, energy yield, and biomass is crucial for effective crop management and resource allocation in plant production. These findings are in line with those of Cotrozzi et al. (2020) [82] and Crusiol et al. (2022a) [11], who reported that PLSR models based on hyperspectral data could accurately predict crop phenotypic parameters in alignment with crop science (Table 2 and Figure 8). The success of these models confirms our initial hypothesis and underscores the potential of using PLSR with hyperspectral data in other agricultural applications and crop management strategies.

### 3.4. Interpolation and Hyperspectral Vegetation Index Analyses for Biophysical and Morphological Parameters

The analysis of interpolation and correlation between biophysical and morphological parameters using hyperspectral reflectance represents a significant advancement in monitoring biomass accumulation in tobacco plants (Figure 9 and Figure 10). Specifically, distinct combinations of hyperspectral bands based on leaves are necessary to monitor each stage of growth and development [12,75,83,84]. The NIR/SWIR band combination was found to be the most effective for predicting height and leaf area, while the VIS−NIR combination was best suited for predicting yield energetic. In consensus with Rodrigues et al. (2022) [15], the VIS−VIS band displayed the highest correlation for biomass. Accordingly, Hassanzadeh et al. (2020) [85] and previous studies [3,5,12,86] have shown that NIR−SWIR bands are effective for predicting plant height and leaf area, while VIS−NIR bands have been used to predict numerous biophysical, physiological, and metabolic attributes and yield energetic in tissues. Thus, it may be possible that the interaction of light with matter influences the differentiation of cellular structures in plants, particularly when these plants are under the influence of GA_3_ and light exposure. This corroborates our initial hypotheses, allowing for the evaluation of morphological parameters using hyperspectral reflectance in SWIR bands.

Interestingly, the shortwave infrared (SWIR) spectrum displayed numerous band associations with increased correlations (>0.61), indicating its potential for accurately predicting plant growth parameters when GA_3_ levels and light fluctuations in environments [13,20,87], although not directly related to greenhouses. Previous studies have also found the SWIR region useful for predicting plant water content, stress, and nutrient uptake [61,86,88]. Furthermore, these band selections were the most responsive wavelengths for tobacco plants based on a selected wavelength (Figure 8, Figure 9, Figure 10 and Figure 11). Consequently, our results showed that the *s*−PLS and *i*−PLS algorithms were more effective in selecting the most responsive wavelengths for various plant characteristics (Figure 10). Specifically, the *s*−PLS algorithm was most significant for SWIR1 and SWIR2, while the *i*−PLS algorithm demonstrated a more uniform distribution of selected wavelengths [8,75], as well as a wavelength_1_ vs. wavelength_2_ by HVI analyses (Figure 11A–D).

Therefore, our study highlights the importance of selecting sensitive spectral bands and using *s*−PLS and *i*−PLS algorithms for accurate plant phenotypic assessments [8,84,89]. Additionally, the SWIR spectrum’s potential to predict plant growth parameters makes it valuable for monitoring plant health and productivity [8]. Overall, these insights underscore the transformative potential of hyperspectral remote sensing to revolutionize plant characteristic prediction and monitoring (Figure 11). Effective band selection can be achieved through XYZ selection or HVI algorithms, correlating with wavelengths selected by *x*−PLS and the vegetation indices proposed in our study. Leveraging these insights can enhance agricultural productivity and sustainability, contributing to a brighter future in crop production management.

### 3.5. Interaction of Light with Biophysical and Morphological Parameters in Leaves

Leveraging the full spectrum from 350 to 2500 nm offers a powerful method for the non-invasive evaluation, classification, and prediction of biophysical parameters through reflectance hyperspectroscopy. This technique could be extended to imaging with hyperspectral or multispectral sensors, including filters in the SWIR1 and SWIR2 regions, to separate the spectral bands [38,58,79,80].

The spectrum captures many leaf components that are essential for biochemical and photosynthetic processes. These include leaf pigments such as chlorophylls, carotenoids, anthocyanins, and flavonoids, as well as anatomical and ultrastructural components [12,17,84]. Leaf structures in the shortwave infrared region can be represented through the spectrum, reflecting the water content, but also cellular structures such as vacuoles, cell walls, and vesicles filled with various molecular compounds [5,7,12,17,20,84].

Therefore, the spectrum provides a comprehensive understanding of leaf production, extending beyond mere pigments to encompass texture, structures, and metabolic activities [5,7,11,12,17,20,84,87]. These emerge from the interaction within cells and leaf tissues, as well as the integration of all spectral bands from 350 to 2500 nm. Consequently, the spectrum can provide valuable insights into biophysical parameters, facilitating evaluations at both the canopy and individual leaf levels.

## 4. Materials and Methods

### 4.1. Experimental Design and Growth Conditions

*Nicotiana tabacum* L. (cv. HAV 425) plants were grown in greenhouses under controlled lighting conditions. Two distinct light intensities were employed, i.e., full (100%) irradiation and low light (8.5% of full irradiance), to investigate their growth parameter responses. The plants were grown in 5 L pots filled with commercial potting mix. A group of plants was treated with gibberellic acid (GA_3_) at different concentrations, and paclobutrazol (PAC) was applied to the soil to observe the effect on plant growth and inhibition of GA metabolism. All the plants were grown under these conditions for 20 days. The spraying regime included five applications every two days, using reverse osmosis water, for the controls (Cont), and 10 µM GA_3_ (GA10), and 100 µM GA_3_ (GA100). PAC 50 mg L^−1^ (PAC) was applied as per the Falcioni et al. (2017) method [20] and combined with GA_3_ as follows: GA_3_ 10 µM + PAC (GA10P) and GA_3_ 100 µM + PAC (GA100P). The experimental design consisted of a 2 × 6 factorial scheme (light vs. GA_3_) with 12 treatments and 6 repetitions for each treatment, totaling 144 samples.

The treatments were designated as follows: CONT (Control; full light), GA10 (10 µM GA_3_; full light), GA100 (100 µM GA_3_; full light), PAC (50 mg L^−1^ of paclobutrazol; full light), GA10P (combination of GA_3_ 10 µM + PAC; full light), GA100P (combination of GA_3_ 100 µM + PAC; full light), SCONT (Control; shade), SGA10 (10 µM GA_3_; shade), SGA100 (100 µM GA_3_; shade), SPAC (50 mg L^−1^ of paclobutrazol; shade), SGA10P (combination of GA_3_ 10 µM + PAC; shade), and SGA100P (combination of GA_3_ 100 µM + PAC; shade) following Falcioni et al. (2017) [20].

### 4.2. Morphological Parameters of Yield and Efficiency of Plants

The growth parameters of yield, such as height (cm), were measured using a graduated ruler from the plant base to the apex. Dry biomass (g) was measured using an analytical balance after desiccating the plant material in a forced-air oven maintained at 70 °C.

### 4.3. Leaf Area Measure

To measure the leaf area (m^2^), a non-destructive analysis was performed by applying the allometric equation following Antunes et al. (2017) [90]. The equation used to estimate the leaf area was [LA = *k* × L × W], where LA represents the leaf area, *k* is a correction factor (0.70014), and maximum length (L) and width (W) for leaves.

### 4.4. Determination of the Yield Energetic of Light for Plants

The leaf area index (LAI) was determined by applying the leaf area (LA) estimation model developed by [90] and by projecting the area on the ground that is covered ($LAI=\frac{LA}{{\pi r}^{2}}$), with the largest leaf length being used as the radius (*r*). To calculate the leaf area volumetric density ($LAD=\frac{LA}{plantvolume}$) for the yield energetic attribute, the aerial part of the plant was considered as a cone projection using the equation $Vcone=\pi r^{2}h$, where (h) represents the length of the stem, resulting in the relationship between the plant’s leaf area and volume (leaf cm^2^ plant cm^−3^) by yield energetic [13].

### 4.5. Reflectance of Hyperspectral Measurements

Hyperspectral reflectance of leaves as measured using a FieldSpec^®^ 3 (Analytical Spectral Devices ASD Inc., Boulder, CO, USA) spectroradiometer and an ASD contact PlantProbe^®^ (Analytical Spectral Devices ASD Inc., Longmont, CO, USA). The spectroradiometer, equipped with three sensors, captured wavelengths from 350 to 2500 nm. The PlantProbe^®^ (Analytical Spectral Devices ASD Inc; USA) ensured that data were free from atmospheric influence. Measurements were taken from the adaxial face of the leaves, excluding the central nervure. Calibration of the spectroradiometers was performed using typical white reference plate standards (Spectralon^®^, Labsphere Inc., Longmont, CO, USA), generating 2151 bands within the 350–2500 nm range [84]. From this procedure, 144 hyperspectral leaf curves were collected, which were subsequently correlated with plant parameters. Specific bands for estimating growth light conditions, gibberellin regime, and leaf colors were selected based on Pearson’s coefficient correlations (*p* < 0.001) and were used to select specific bands for estimating parameters and generating a cluster heatmap.

### 4.6. Statistical Analyses

#### 4.6.1. Descriptive and Univariate Statistical Analyses

Descriptive statistics for growth parameters, including mean, standard error, maximum, minimum, and coefficient of variation (CV, %), were calculated as per [91], with CV classified based on [7]. One−way ANOVA was conducted to compare the means of growth parameters, with statistical significance set at *p* < 0.001 [92], and a pairwise comparison of means was performed using Duncan’s test, also at *p* < 0.001. Pearson’s correlation test was applied to investigate relationships between the growth parameters and vegetation indices. Statistical analyses were performed by Statistica 10^®^ software (Statsoft Inc., Tulsa, OK, USA). Graphs were generated using SigmaPlot 10.0^®^ (Systat Inc., Santa Clara, CA, USA) and CorelDraw 2020^®^ (Corel Corp., Ottawa, ON, CAN).

#### 4.6.2. Principal Component Analysis (PCA)

PCA was performed on the growth parameter data using The Unscrambler X software, version 10.4 (CAMO Software, Oslo, NOR), with statistical significance set at *p* < 0.001. To prevent underfitting and overfitting, the optimal number of principal components was selected based on the first maximum value of the overall accuracy. The resulting base PCAs were classified according to [37].

#### 4.6.3. Machine Learning and Artificial Intelligence Algorithm (AIA) Models

Eight different AI algorithms for machine learning and data mining were employed. These algorithms were performed by Orange Data Mining 3.33 (Open−Software). The algorithms used include neural network (NN), gradient boosting (GB), random forest (RF), support vector machine (SVM), kernel k-nearest neighbors (KNN), naive Bayes (NB), logistic regression (LR), and stochastic gradient descent (SGD), as detailed in Table S1. The evaluated data were randomly separated into 70% for training and 30% for testing. The results were analyzed based on precision and recall data, and the algorithms’ performance was evaluated using a confusion matrix. Graphs were generated using CorelDraw, and an online platform for generation and visualization (https://www.bioinformatics.com.cn/en, accessed on 26 May 2023) was used to plot the data.

#### 4.6.4. Vegetation Indices Analyses

The study involved testing each of the vegetation indices under different growth conditions of full light and shade, as well as varying regimes of gibberellin. Furthermore, the relative performance and contribution of the vegetation indices were compared in monitoring the plant’s status, including photosynthetic pigments, photochemical efficiency, water status, pigment content, and structural changes. The results of the vegetation indices analysis are presented in Table S1 and Figure S1.

#### 4.6.5. Analysis of Reflectance Non-Imaging Sensors Using Partial Least Squares Regression (PLSR)

The dataset was divided into two groups: one comprising 100 samples for calibration and cross-validation and the other consisting of 44 independent samples for external prediction of the PLSR model. Plant growth parameters, including height (cm), leaf area (m^2^), yield energetic (m^3^), and biomass (g), were compared to the spectral curves, treating each variable as an independent factor. The PLSR models were constructed using the NIPALS algorithm, and outlier limits were determined based on Leverage’s type and analyzed using Leverage and Hotelling’s T^2^ (with a limit of 5%). To evaluate the predictive performance of the models, coefficients of determination (R^2^) and root mean square error (RMSE) were calculated for calibration, cross-validation, and prediction phases. Following the criteria proposed by Minasny and McBratney (2013) [93], R^2^ values greater than 0.75 were considered excellent predictors, values between 0.75 and 0.5 were considered good, and values below 0.5 were regarded as low. Moreover, the ratio of performance to deviation (RPD) was calculated using R^2^ for calibration (R^2^_C_), cross-validation (R^2^_CV_), and predicted (R^2^_P_) as an informative indicator of the expected accuracy of PLS predictions, as described in [73].

#### 4.6.6. Comparison of Wavelength_1_ vs. Wavelength_2_ for Improved Monitoring of Plant Growth Parameters

To evaluate whether selecting specific hyperspectral bands can enhance the accuracy of monitoring plant growth parameters, we conducted a study using Pearson’s correlation. We evaluated all possible combinations of two spectral bands, with each combination representing a single hyperspectral band that we assessed for height, leaf area, yield energetic, and biomass at various wavelengths using the Pearson correlation coefficient (r) and coefficient of determination (R^2^). We performed an analysis of the hyperspectral sensor using the full spectrum ranging from 350 to 2500 nm and applied an inverse distance to a power algorithm to ensure a fair comparison between the hyperspectral bands. Surfer Software version 22 (Golden Software; Golden, CO, USA) was utilized to analyze the results. A planar regression was applied using the equation Z = AX + BY + C, and the analysis was conducted using the XYZ statistic with the contour map to compare the selected hyperspectral bands and their corresponding attributes. This approach allowed us to determine whether selecting specific hyperspectral bands could enhance the accuracy of monitoring morphological characteristics and yields for plant growth.

#### 4.6.7. Phenotypic Parameter Assessment through Variable Selection Algorithms

Six algorithms were employed for variable selection: variable importance in projection (VIP), genetic algorithm (GA), sparse partial least squares regression (*s*−PLS), interval partial least squares regression (*i*−PLS), recursive partial least squares regression (*r*−PLS), and nonlinear partial least squares regression (*n*−PLS) [8]. These algorithms were utilized to identify and select the most responsive wavelengths within the 350 to 2500 nm range for evaluating the phenotypic parameters of tobacco plants. Data analysis was conducted using MATLAB 2022a software (MathWorks, Inc., Natick, MA, USA) and PLS_Toolbox (Eigenvector Research, Inc., Manson, WA, USA). The efficacy of each algorithm was assessed based on its capacity to differentiate between wavelengths and select the most responsive ones for generating phenotypic classification models related to height (cm), leaf area (m^2^), yield energetic (m^3^), and biomass (g).

#### 4.6.8. Hyperspectral Vegetation Index for Phenotyping Tobacco Plants

To evaluate the potential enhancement in phenotyping accuracy through the selection of two optimal narrow hyperspectral bands, a comprehensive analysis of all possible combinations of two spectral bands was conducted using the normalized difference vegetation index (NDVI) formula (Equation (1)), as suggested by Crusiol et al. (2023) [94] and Crusiol et al. (2022b) [67]. Each combination, representing a specific hyperspectral vegetation index (HVI), was subsequently correlated with phenotyping efficiency parameters using the Pearson correlation coefficient (r) and the coefficient of determination (R^2^). The calculations were performed using custom-created code in the IDL language. The ground-based sensor captures the complete spectra ranging from 350 nm to 2500 nm. The results are visually represented as contour maps.

(1) $$HVI=\frac{Wavelength1-Wavelength2}{Wavelength1+Wavelength2}$$

## 5. Conclusions

The findings of this study demonstrate the substantial potential of reflectance hyperspectral technology in precision and digital agriculture. It has been effectively used in phenotyping, classifying, and predicting growth parameters of tobacco plants under varied light conditions and gibberellin levels. The applicability of this methodology extends beyond tobacco, suggesting possible implementation for monitoring growth in other agricultural crops within greenhouses. This technology, therefore, holds promise for enhancing agricultural productivity and sustainability by providing accurate and efficient methods for crop growth and development. Furthermore, the emphasis on the development of alternative methodologies for rapid plant characteristic quantification aligns with the anticipated progression of digital and precision remote sensing technology, indicating a bright future for research and innovation in this field.

## Figures and Tables

**Table 1:** Statistical description of biophysical and efficiency parameters in *Nicotiana tabacum* L. plants.

Parameters	Count (n)	Mean	Median	Minimum	Maximum	CV (%)
Height (cm)	144	77.4	73.9	30.2	142.5	33.5
Leaf area (m^2^)	144	0.3	0.3	0.1	0.8	51.8
Yield energetic (m^3^)	144	9.6	8.8	1.3	29.5	59.7
Biomass (g)	144	21.3	11.4	2.5	55.1	82.6

**Table 2:** Statistical parameters obtained for *Nicotiana tabacum* L. plants during the calibration, cross-validation, and validation phases of the unspoked state models. (*n* = 144).

PLSR Models	Attributes	PLSR Parameters						
		Factors	r	R^2^	Offset	RMSE	RPD	Bias
**Calibration**	Height (cm)	3	0.93	0.86	10.6	9.4	2.70	−
	Leaf area (m^2^)	3	0.91	0.83	0.1	0.1	2.45	−
	Yield energetic (m^3^)	1	0.93	0.86	1.8	2.3	2.67	−
	Biomass (g)	3	0.94	0.88	2.4	6.1	2.94	−
**Cross-Validation**	Height (cm)	3	0.92	0.84	11.6	10.2	2.52	−
	Leaf area (m^2^)	3	0.90	0.81	0.1	0.1	2.30	−
	Yield energetic (m^3^)	1	0.91	0.84	2.0	2.5	2.48	−
	Biomass (g)	3	0.93	0.87	2.7	6.6	2.73	−
**Prediction**	Height (cm)	3	0.88	0.77	15.0	12.2	2.09	1.6
	Leaf area (m^2^)	3	0.89	0.79	0.1	0.1	2.18	0.0
	Yield energetic (m^3^)	1	0.90	0.81	1.9	2.4	2.31	0.2
	Biomass (g)	3	0.93	0.87	2.6	6.3	2.76	0.3

## Data Availability

The data presented in this study are available in Supplementary Materials.

## Supplementary Materials

The following supporting information can be downloaded at: https://www.mdpi.com/article/10.3390/plants12132526/s1. Table S1. Vegetation indices parameters applied for *Nicotiana tabacum* L. leaves of plants grown in high irradiance (full light) and low light (8.5% of full light) environments and submitted to a distinct gibberellin regime. Figure S1. Vegetation index parameters calculated for arbitrary values (%) on *Nicotiana tabacum* L. leaves of plants grown in high irradiance (full light) and low light (8.5% of full light) environments and submitted to distinct gibberellin regimes. For abbreviations, see Figure 1 and Table S1. References [71,74,78,95,96,97,98,99,100,101,102,103,104] are cited in the Supplementary Materials.
