# Supplementary material for: Non−Invasive Assessment, Classification, and Prediction of Biophysical Parameters Using Reflectance Hyperspectroscopy
Source: Plants (Basel). 2023 Jul 2;12(13):2526. doi: 10.3390/plants12132526 (PMC10347113; doi:10.3390/plants12132526)
Supplement: Supplementary file 1 [file plants-12-02526-s001.zip › plants-2453036-supplementary.pdf]

plants

MDPI

Supplementary Materials

Non−Invasive Assessment, Classification, and Prediction of Biophysical Parameters Using Reflectance Hyperspectroscopy

Renan Falcioni [1,*], Glaucio Leboso Alemparte Abrantes dos Santos [1], Luis Guilherme Teixeira Crusiol [2], Werner Camargos Antunes [1], Marcelo Luiz Chicati [1], Roney Berti de Oliveira [1], José A. M. Demattê [3] and Marcos Rafael Nanni [1]

[1] Department of Agronomy, State University of Maringá, Av. Colombo, 5790, Maringá 87020−900, Paraná, Brazil; glaucioalemparte@gmail.com (G.L.A.A.d.S.); wcantunes@uem.br (W.C.A.); mlchicati@uem.br (M.L.C.); rboliveira@uem.br (R.B.d.O.); mrnanni@uem.br (M.R.N.)
[2] Embrapa Soja (National Soybean Research Center−Brazilian Agricultural Research Corporation), Rodovia Carlos João Strass, s/nº, Distrito de Warta, Londrina 86001–970, Paraná, Brazil; luis.crusiol@colaborador.embrapa.br
[3] Department of Soil Science, Luiz de Queiroz College of Agriculture, University of São Paulo, Av. Pádua Dias, 11, Piracicaba 13418−260, São Paulo, Brazil; jamdemat@usp.br
* Correspondence: renanfalcioni@gmail.com; Tel.: +55-4430118940

Table S1. Vegetation indices parameters applied for *Nicotiana tabacum* L. leaves of plants grown in high irradiance (full light) and low light (8.5% of full light) environments and submitted to a distinct gibberellin regime.

Index	Equation	Reference
$NDVI_{680}$ = Normalized Difference Vegetation Index ρ680	$(R_{800}-R_{680})/(R_{800}+R_{680})$	[71]
$NDVI_{750}$ = Normalized Difference Vegetation Index ρ750	$(R_{750}-R_{705})/(R_{750}+R_{705})$	[71]
SR_{680} = Simple Ratio Index ρ680	(R_{800}/R_{680})	[71]
SR_{705} = Simple Ratio Index ρ705	$(R_{750})/(R_{705})$	[95]
mSR_{705} = Modified Normalized Simple Ratio ρ705	$(R_{750}-R_{445})/(R_{705}+R_{445})$	[96]
$mNDVI_{750}$ = Modified Normalized Difference Vegetation Index ρ750	$(R_{750}-R_{705})/(R_{750}+R_{705} - 2xR_{445})$	[97]
RARS = Ratio Analysis of Reflectance Spectra	$(R_{746})/(R_{513})$	[98]
Achl = Absorption of Chlorophyll Index	$(R_{550})/(R_{500})$	[99]
BNb = Index for Chlorophyll Content	$(R_{800})/(R_{550})$	[99]
PVR = Normalized Difference Photosynthetic	$(R_{550}-R_{650})/(R_{550}+R_{650})$	[97]
PSND = Pigment Specific Normalized Difference	$(R_{800}-R_{470})/(R_{800}+R_{470})$	[100]
PSSRa = Pigment Specific Simple Ratio Chl *a*	$(R_{800})/(R_{680})$	[98]
PSSRb = Pigment Specific Simple Ratio Chl *b*	$(R_{800})/(R_{635})$	[95]
PSSRc = Pigment-specific Simple Ratio	$(R_{800})/(R_{500})$	[95]
PSRI = Plant Senescence Reflectance Index	$(R_{680}-R_{500})/(R_{750})$	[97]
PSRI2 = Plant Senescence Reflectance Index 2	$(R_{672})/(R_{550}+R_{708})$	[97]
MSI = Moisture Stress Index	(R_{1650}/R_{830})	[99]
PRI = Photochemical Reflectance Index	$(R_{530}-R_{570})/(R_{530}+R_{570})$	[101]
FR = Fluorescence Ratio	$(R_{690})/(R_{740})$	[102]
WBI = Water Band Index	$(R_{900})/(R_{970})$	[103]
DSWI = Disease-Water Stress Index	$(R_{802}+R_{547})/(R_{1657}+R_{682})$	[97]
DSWI-5 = Disease-Water Stress Index 5	$(R_{800}-R_{550})/(R_{1660}+R_{680})$	[97]

CRI1 = Carotenoid Reflectance Index 1	$(1/R_{510})$-$(1/R_{550})$	[98]
CRI2 = Carotenoid Reflectance Index 2	$(1/R_{510})$-$(1/R_{700})$	[71]
ARI1 = Anthocyanin Reflectance Index	$(1/R_{550})$-$(1/R_{700})$	[100]
ARI2 = Anthocyanin Reflectance Index 2	R_{800} x $((1/R_{550})$-$(1/R_{700}))$	[71]
FRI = Flavonol Reflectance Index	R_{800} x $((1/R_{410})$-$(1/R_{460}))$	[99]
VOG1 = Vogelmann Index 1	$(R_{740})/(R_{720})$	[99]
VOG2 = Vogelmann Index 2	$(R_{734}$-$R_{747})/(R_{715}+R_{726})$	[100]
SIPI = Structurally Insensitive Pigment Index	$(R_{800}$-$R_{445})/(R_{800}$-$R_{680})$	[104]
CAI1 = Cellulose Absorption Index 1	100 x $(0.5(R_{2030}+R_{2210})$-$R_{2100}))$	[74]
CAI2 = Cellulose Absorption Index 2	0.5 x $(R_{2020}+R_{2220})$-$R_{2100}))$	[74]
NDLI = Normalized Difference Lignin Index	$[\log(1/R_{1754})$-$\log(1/R_{1680})]/[\log(1/R_{1754})+\log(1/R_{1680})]$	[78]
NDNI = Normalized Difference Nitrogen Index	$[\log(1/R_{1510})$-$\log(1/R_{1680})]/[\log(1/R_{1510})+\log(1/R_{1680})]$	[78]

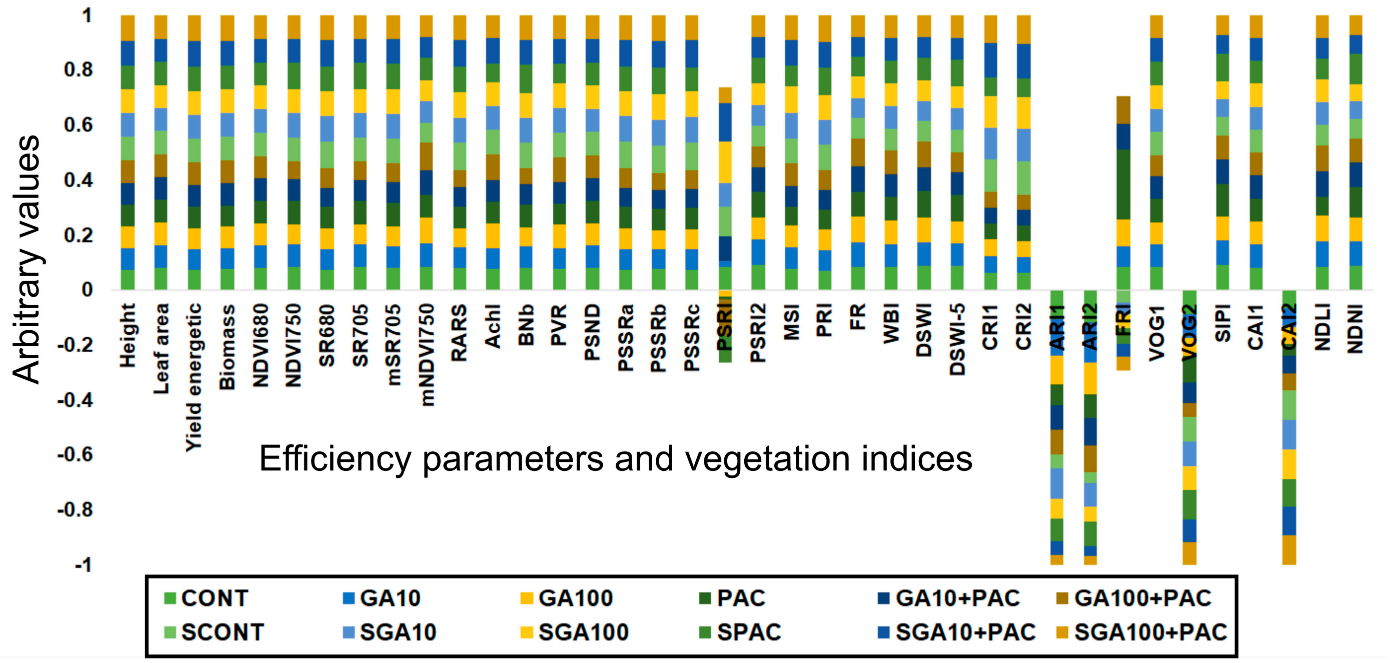

Figure S1. Vegetation index parameters calculated for arbitrary values (%) on *Nicotiana tabacum* L. leaves of plants grown in high irradiance (full light) and low-light (8.5% of full light) environments and submitted to distinct gibberellin regimes. For abbreviations, see Figure 1 and Table S1.

References

71. Gitelson, A.; Merzlyak, M.N. Spectral Reflectance Changes Associated with Autumn Senescence of *Aesculus hippocastanum* L. and *Acer platanoides* L. Leaves. Spectral Features and Relation to Chlorophyll Estimation. *J. Plant Physiol.* **1994**, 143, 286–292.
74. Nagler, P.L.; Inoue, Y.; Glenn, E.P.; Russ, A.L.; Daughtry, C.S.T. Cellulose Absorption Index (CAI) to Quantify Mixed Soil–Plant Litter Scenes. *Remote Sens. Environ.* **2003**, 87, 310–325.
78. Serrano, L.; Peñuelas, J.; Ustin, S.L. Remote Sensing of Nitrogen and Lignin in Mediterranean Vegetation from AVIRIS Data: Decomposing Biochemical from Structural Signals. *Remote Sens. Environ.* **2002**, 81, 355–364.

95. Lang, M.; Stober, F.; Lichtenthaler, H.K. Fluorescence Emission Spectra of Plant Leaves and Plant Constituents. Radiat. *Environ. Biophys.* **1991**, 30, 333–347.
96. Stimson, H.C.; Breshears, D.D.; Ustin, S.L.; Kefauver, S.C. Spectral Sensing of Foliar Water Conditions in Two Co-Occurring Conifer Species: *Pinus edulis* and *Juniperus monosperma*. *Remote Sens. Environ.* **2005**, 96, 108–118.
97. Apan, A.; Held, A.; Phinn, S.; Markley, J. Formulation and Assessment of Narrow-Band Vegetation Indices from EO-1 Hyperion Imagery for Discriminating Sugarcane Disease. In Proceedings of the 2003 Spatial Sciences Institute Biennial Conference: Spatial Knowledge Without Boundaries (SSC2003), Canberra, Australia, 22–27 September 2003; pp. 1–13.
98. Chappelle, E.W.; Kim, M.S.; McMurtrey, J.E. Ratio Analysis of Reflectance Spectra (RARS): An Algorithm for the Remote Estimation of the Concentrations of Chlorophyll A, Chlorophyll B, and Carotenoids in Soybean Leaves. *Remote Sens. Environ.* **1992**, 39, 239–247.
99. Pontius, J.; Martin, M.; Plourde, L.; Hallett, R. Ash Decline Assessment in Emerald Ash Borer-Infested Regions: A Test of Tree-Level, Hyperspectral Technologies. *Remote Sens. Environ.* **2008**, 112, 2665–2676.
100. Metternicht, G. Vegetation Indices Derived from High-Resolution Airborne Videography for Precision Crop Management. *Int. J. Remote Sens.* **2003**, 24, 2855–2877.
101. Blackburn, G.A. Spectral Indices for Estimating Photosynthetic Pigment Concentrations: A Test Using Senescent Tree Leaves. *Int. J. Remote Sens.* **1998**, 19, 657–675.
102. Merzlyak, M.N.; Chivkunova, O.B.; Solovchenko, A.E.; Naqvi, K.R. Light Absorption by Anthocyanins in Juvenile, Stressed, and Senescing Leaves. *J. Exp. Bot.* **2008**, 59, 3903–3911.
103. Gitelson, A. Nondestructive Estimation of Foliar Pigments (Chlorophylls, Carotenoids, and Anthocyanins) Contents: Evaluating a Semianalytical Three-Band Model. In Hyperspectral Remote Sensing of Vegetation; Thenkaail, P.S., Lyon, J.G., Huete, A., Eds.; CRC Press: New York, NY, USA, 2011; p. 782.
104. Garbulsky, M.F.; Peñuelas, J.; Gamon, J.; Inoue, Y.; Filella, I. The Photochemical Reflectance Index (PRI) and the Remote Sensing of Leaf, Canopy and Ecosystem Radiation Use Efficiencies. A Review and Meta-Analysis. *Remote Sens. Environ.* **2011**, 115, 281–297.
